# Migraine in gulf war illness and chronic fatigue syndrome: prevalence, potential mechanisms, and evaluation

**DOI:** 10.3389/fphys.2013.00181

**Published:** 2013-07-24

**Authors:** Rakib U. Rayhan, Murugan K. Ravindran, James N. Baraniuk

**Affiliations:** Division of Rheumatology, Immunology and Allergy, Department of Medicine, Georgetown UniversityWashington, DC, USA

**Keywords:** migraine, gulf war illness, chronic fatigue syndrome, fibromyalgia, central sensitization, chronic pain, fatigue, neurolimbic pathway

## Abstract

**Objective:** To assess the prevalence of headache subtypes in Gulf War Illness (GWI) and Chronic Fatigue Syndrome (CFS) compared to controls.

**Background:** Approximately, 25% of the military personnel who served in the 1990–1991 Persian Gulf War have developed GWI. Symptoms of GWI and CFS have considerable overlap, including headache complaints. Migraines are reported in CFS. The type and prevalence of headaches in GWI have not been adequately assessed.

**Methods:** 50 GWI, 39 CFS and 45 controls had structured headache evaluations based on the 2004 International Headache Society criteria. All subjects had history and physical examinations, fatigue and symptom related questionnaires, measurements of systemic hyperalgesia (dolorimetry), and assessments for exclusionary conditions.

**Results:** Migraines were detected in 64% of GWI (odds ratio = 11.6 [4.1–32.5]) (mean [±95% CI]) and 82% of CFS subjects (odds ratio = 22.5 [7.8–64.8]) compared to only 13% of controls. There was a predominance of females in the CFS compared to GWI and controls. However, migraine status was independent of gender in GWI and CFS groups (*x*^2^ = 2.7; *P* = 0.101). Measures of fatigue, pain, and other ancillary criteria were comparable between GWI and CFS subjects with and without headache.

**Conclusion:** The high prevalence of migraine in CFS was confirmed and extended to GWI subjects. GWI and CFS may share dysfunctional central pathophysiological pathways that contribute to migraine and subjective symptoms. The high migraine prevalence warrants the inclusion of a structured headache evaluation in GWI and CFS subjects, and treatment when present.

## Introduction

Gulf War Illness (GWI), also known as Chronic Multisymptom Illness (CMI), affects 25–30% of the 697,000 veterans deployed to the 1990–1991 Persian Gulf War (Fukuda et al., 1998; Gray et al., 2002; RAC-GWVI, 2008; Li et al., 2011). Veterans with GWI suffer from a wide array of symptoms that include headaches, cognitive dysfunction, chronic pain, fatigue, and other complaints (Fukuda et al., 1998; Gray et al., 2002; Li et al., 2011). Gulf War veterans often meet the criteria for chronic fatigue syndrome (CFS) (Fukuda et al., 1994) and fibromyalgia (FM) strongly suggesting overlap in symptoms for GWI and CFS (Wolfe et al., 1990; Peres et al., 2002; Latremoliere and Woolf, 2009; Baraniuk and Zheng, 2010; Wolfe et al., 2010; Ravindran et al., 2011; de Tommaso et al., 2011).

Headache is one of the eight ancillary criteria in the CFS case definition (Fukuda et al., 1994; Baraniuk and Zheng, 2010; Ravindran et al., 2011). Complaints of headaches are also reported in Gulf War veterans and over 50% of FM patients (Gray et al., 2002; de Tommaso et al., 2011; Li et al., 2011). However, the type and severity of headaches are not evaluated as a part of the GWI, CFS, or FM case designation criteria (Wolfe et al., 1990; Fukuda et al., 1994, 1998; RAC-GWVI, 2008; Wolfe et al., 2010; Ravindran et al., 2011). We recently determined that migraines are the primary type type of headaches in CFS (Ravindran et al., 2011). Studies in FM have reported similar migraine predominance (de Tommaso et al., 2011). The exact relationships between headache subtypes, fatigue, pain, hyperalgesia, and other systemic complaints has been difficult to establish because of heterogeneous symptom presentation and/or lack of biomarkers that identify a distinct pathophysiological process in GWI.

One mechanism that has been proposed to drive the complaints of GWI and related illnesses is central sensitization (Latremoliere and Woolf, 2009; Baraniuk and Zheng, 2010). This process is defined as an amplification of responsiveness of central pain-signaling neurons that clinically manifest as hyperalgesia and/or allodynia (Latremoliere and Woolf, 2009). This normal adaptive neuronal process is essential for learning, but has been proposed to become dysregulated in each of these disorders and in even in migraineurs (Gebhart, 2004; DaSilva et al., 2007; Tietjen et al., 2009).

Neuroimaging studies report underlying dysfunction in brain energetics, the brainstem, and thalamocortical tracts in migraineurs (DaSilva et al., 2007). Alterations in these regions may lead to the loss of descending anti-nociceptive processes coupled with changes in cerebrovascular dynamics. Specifically, blood flow alterations in migraine headache are associated with dysfunction in the trigeminovascular system, which innervate cerebral blood vessels in the dura (Karatas et al., 2013). As a result of trigeminal activation, migraineurs demonstrate hyperalgesia, allodynia, and cognitive dysfunction during and between episodes (Gebhart, 2004; DaSilva et al., 2007; Tietjen et al., 2009; Baraniuk and Zheng, 2010; Karatas et al., 2013). These findings may lend credence to the theory of central sensitization.

**Figure 1:**
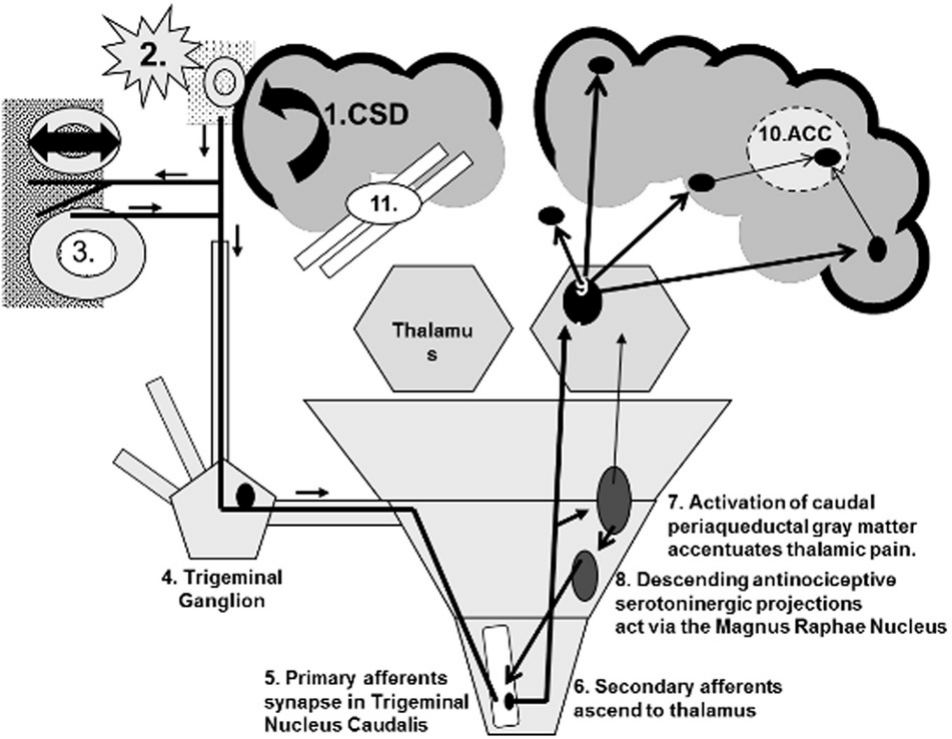
**Central sensitization using migraine as a model.** (1) Cortical spreading depression (CSD) depolarizes cortical neurons and glia. (2) They release glutamate, K+, H+, metalloproteases, and other agents that dilate pial vessels and activate trigeminal nociceptive nerves. (3) The bifurcated neurons release calcitonin gene related peptide (CGRP) and other vasodilators near dural vessels by the axon response mechanism. Vascular wall stretching activates additional trigeminal nociceptive neurons (4) that have their primary synapse (5) in the upper cervical dorsal horn. (6) Ascending secondary afferents activate the thalamus. (7) Other afferents signal the periaqueductal gray matter. (8) Descending relays to the magnus raphae nucleus activate descending serotonergic neurons to inhibit the primary trigeminal (5 and 6) synapses. (9) Thalamocortical projections stimulate the hypothalamus, somatosensory cortex, amygdala, limbic system, and frontal cortex. (10) Pain, emotion, memory, frontal processing and other inputs converge on the anterior cingulate gyrus (ACC) and (11) interfere with its executive decision-making functions.

Similar patterns of gray and white matter abnormalities and altered brain energetics in GWI, CFS, FM, and migraine suggest that common central mechanisms may contribute to the type of headaches and the cognitive impairments perceived as “brain fog” (Lutz et al., 2008; Barnden et al., 2011; Puri et al., 2012; Rayhan et al., 2013a,b). Understanding the pathophysiological mechanisms underlying migraine may identify the primary dysfunction that leads to the constellation of complaints in GWI, CFS, and other overlapping disorders (Figure 1).

Although GWI patients complain of headache symptoms that overlap with CFS, FM, and migraineurs, there is currently no study that specifically assesses this in GWI. We hypothesized that due to extensive symptom overlap, GWI subjects will have similar rates of headache complaints subtypes. As a first step toward testing this hypothesis, we focused on identifying clinical relationships between headache status, hyperalgesia (tenderness), pain, and fatigue ratings in GWI. In addition, we compared such complaints to CFS subjects and controls to further identify similarities and/or differences between groups. The cross-sectional design permitted estimation of the prevalences of headache, pain and fatigue in GWI. Comparisons of GWI to CFS subjects allowed validation and extension of previous findings. Migraine status, GWI, CFS, FM co-morbidity and other conditions were diagnosed based on history and physical examination rather than mail-in and/or online questionnaires. Detailed history and physical examination has proved to be highly reliable in previous studies (Ravindran et al., 2011). To supplement the research study, we review the current literature into migraines and potential mechanisms.

## Materials and methods

### Subjects

GWI, CFS, and sedentary control subjects were recruited in 2 consecutive cohorts and combined for this analysis. Both cohorts had populations of all three groups (GWI, CFS, and controls). Cohort 1 was recruited under Georgetown University Institutional Review Board protocol #2006-481 (clinicaltrials.gov identification number NCT00810329) between 2007 and 2009. Cohort 2 participated in a separate protocol between 2009 and 2011 that was approved by the Georgetown University Institutional Review Board (#2009-229; clinicaltrials.gov NCT01291758) and United States Army Medical Research Materiel Command Human Research Protection Office (USAMRMC HRPO #A-15547.0).

Approximately 400 GWI, CFS, and sedentary control subjects responded to print and on-line advertising by contacting our laboratory by telephone or e-mail. Each volunteer had an initial telephone screening during which they gave verbal consent. Cohort 1 had 124 total eligible subjects identified after telephone screening. Of the 124, 83 particpants completed the overall study. Cohort 2 had 167 total eligible subjects identified after telephone screening. Of the 167, 83 participants completed the overall study. The 41 and 116 of the eligible participants from Cohort 1 and 2, respectively, who were identified to be eligible after telephone screening o did not participate due to lack of response to follow-up emails, phone calls, or did not show up to the scheduled clinic visit.

Upon arrival in the Clinical Research Unit, all participants who decided to complete the study reviewed and signed their informed consent forms. All subjects had history and physical examinations with scripted interviews to assess migraine, tension headache, CMI, CFS, and FM criteria.

### Inclusion and case designation criteria

Inclusion criteria for veterans included military service for at least 30 days between August 1, 1990 and July 31, 1991. GWI was designated using the 1998 Center for Disease Control (CDC) criteria for CMI (Fukuda et al., 1998) with deployment for ≥30 consecutive days to the Persian Gulf War region (RAC-GWVI, 2008). CMI criteria required >6 months of complaints from at least two of the following categories: (i) fatigue; (ii) musculoskeletal pain (muscle pain, joint pain, stiffness of joints); and (iii) cognition and mood (feeling depressed, trouble remembering or focusing, mood changes, anxious feelings, difficulty finding words, and difficulty sleeping) (Fukuda et al., 1998).

CFS was defined by the 1994 CDC criteria (Fukuda et al., 1994). Fatigue lasting >6 months with no medical, psychiatric, or other attributable cause was required along with at least 4 of the 8 ancillary criteria: (i) cognitive problems with memory and concentrating, (ii) sore throat, (iii) sore lymph node regions, (iv) myalgia, (v) arthralgia, (vi) headache with onset after the fatigue, (vii) sleep disturbances with un-refreshing sleep, or (viii) significant exacerbations of the severe fatigue and the other symptoms immediately or as long as 24 h following exercise, cognitive, or another activity that was more strenuous than usual. All GWI subjects were also screened for CFS status during history and physical examination to assess criteria overlap in conditions (Fukuda et al., 1994).

To assess comorbidity in CFS and GWI, FM was also identified using the 1990 criteria (Wolfe et al., 1990) since the study started before the 2010 criteria were introduced (Wolfe et al., 2010). Subjects were assessed for widespread pain in 4 quadrants above and below the waist, to the left and right of the midline and involving the axial skeleton that had been present 3 months and had no explanation. Manual thumb pressure of about 4 kg was applied to 18 traditional tender points. FM required both widespread pain and tenderness at ≥11 of 18 points (Wolfe et al., 1990).

Migraine was defined by International Headache Society criteria (Headache Classification Subcommittee of the International Headache Society, 2004) using a structured interview to determine if a subject had ≥5 episodes lasting 4–72 h with at least 2 of (a) unilateral cephalgia, (b) pulsatile quality, (c) moderate to severe pain severity, and (d) aggravation of the headache by usual activities leading to disinclination to perform usual work or other activities of daily living. Sensitivity to light or sound, or nausea with or without emesis were required. Auras were assessed for their prescient ability. For CFS diagnostic purposes, migraines had to be of new onset coinciding with symptoms of CFS, and so subjects with perimenstrual and progression of headaches with childhood onset were excluded from consideration. Information was collected about the duration of headaches and will be discussed in an alternate manuscript.

Exclusion criteria included positive pregnancy test, lactation, active duty military personnel, claustrophobia, cardiovascular restrictions, intolerance of needles, ferrous based implants, major psychiatric illness, infectious status (e.g., HIV), neoplasm, untreated endocrine and other chronic disease that may have accounted for GWI or CFS associated symptoms (Reeves et al., 2003, 2005; Sullivan et al., 2005; RAC-GWVI, 2008; Jones et al., 2009).

### Questionnaires

Subjects from cohort 1 completed paper questionnaires. Subjects from cohort 2 received a confidential website log-in identifier, password and identification code so they could complete the pre-study symptom and psychometric questionnaires using our online confidential data collection procedure (Rayhan et al., 2013c). No personal identifying information was collected using either system. Locked facilities were under 24 h surveillance. Workstations were only accessible by approved personnel and protected by 128-bit encrypted password.

Subjects completed the CFS symptom severity questionnaire to self-report their symptoms for the past 6 months (Baraniuk et al., 2013). The severity of fatigue and the 8 ancillary symptoms was assessed using an anchored ordinal scale with 0 = no complaint, 1 = trivial, 2 = mild, 3 = moderate, or 4 = severe intensity. The scores of the 8 ancillary criteria were calculated and the summed and are reported as “Sum8” (Baraniuk et al., 2013).

Subjective pain perceptions were quantified using the McGill short form with its sensory, affective and total scores (Melzack, 1987) and relative disability and quality of life using the Medical Outcomes Survey Short Form 36 (SF-36) (Ware and Sherbourne, 1995).

### Protocol

Hyperalgesia in FM has traditionally been ascertained by tenderness to manual thumb pressure of about 4 kg at ≥11 of 18 tender points (Wolfe et al., 1990). We adapted this concept by pressing a pressure strain gauge dolorimeter at a rate of 1 kg/s at the 18 sites and over 5 paranasal sinus regions on all subjects (Naranch et al., 2002; Rayhan et al., 2013a). Subjects were told that they were in complete control of the pressure, and to report the point when the pressure sensation switched to become painful. The mean of the dolorimetry pressures has been previously used as a measure of systemic and sinus hyperalgesia (Naranch et al., 2002; Rayhan et al., 2013a).

### Statistical analysis

Data were tabulated in Microsoft Excel 2010 (Redmond, Washington) for analysis with SPSS for Windows version 20 (IBM, Armonk, NY). Means were reported with [±95% confidence intervals (CI)]. Significant differences in migraine status and gender between the three groups were identified using the 2 × 3 Fisher's exact probability test with the Freeman-Halton extension and two-tailed *P*-values (Freeman and Halton, 1951). Migraine headaches and CFS are more common in females than males (Lea et al., 2004; Ravindran et al., 2011; Critchley and Harrison, 2013). Due to this, gender was assessed as a controlled comparison to test its influence on migraine headache and GWI and/or CFS status. All variables were entered as dichotomous values (0 or 1) and results from this analysis are reported with continuity correction (Sweeting et al., 2004).

To protect against inflating the type 1 error rate, one way multivariate analysis of variance (MANOVA) was used across all 15 non-dichotomous variables which included the ordinal rankings in the CFS severity score, McGill total pain score and its subscales, and the continuous scale systemic and sinus dolorimetry values. This was followed by One-Way analysis of variance (ANOVA) on each variable with post hoc testing using Tukey's Honest Significant Difference (HSD) test.

## Results

### Demographics

50 GWI, 39 CFS, and 45 control subjects were recruited. All of the GWI subjects also met criteria for CFS. More than half of the GWI and control subjects were male, with a female predominance of 4:1 in CFS (*P* = 0.00001 by Fisher's Exact test), with no significant differences in age (Table 1).

**Table 1:** **Demographics**.

	**Controls (*n* = 45)**	**CFS (*n* = 39)**	**GWI (*n* = 50)**	***P*-value**
Age	43.7 [39.9–47.5]	46.3 [43.1–49.5]	46.7 [44.3–49.1]	
% Male	53.3%	20.5%	68.0%	0.00001^*^

* 2 × 3 Fisher's exact test; Mean [±95% CI].

### Headache status

Migraines were detected in 64% of GWI (odds ratio = 11.6 [4.1–32.5]) and 82% of CFS (odds ratio = 22.5 [7.8–64.8]) subjects compared to 13% of controls (*P* = 5.5 × 10^−11^; Table 2). Migraines status was characterized with aura (MA) and without aura (MO) with results showing the anticipated 3:1 ratio (Table 2). Co-morbid migraine and tension headaches were frequent. Tension headaches without a migraine diagnosis were present in ~20% of GWI, 7% of CFS, and 26% of control subjects (Table 2). Gender did not influence CFS, GWI, and migraine status [χ^2^_(1, *n* = 89)_ = 2.69, *P* = 0.10].

**Table 2:** **Headache status**.

**Parameter**	**Controls**	**CFS**	**GWI**	
	**(*n* = 45)**	**(*n* = 39)**	**(*n* = 50)**	
Migraine headache	6/45 (13.3%)	32/39 (82.1%)	32/50 (64%)	5.5 × 10^−11^^*^
With Aura (MA)	1/6	22/32	24/32	
Without Aura (MO)	5/6	10/32	8/32	
Comorbid migraine and tension headaches	3/6	24/32	20/32	
Tension headache alone	12/45 (26.6%)	3/39 (7.6%)	10/50 (20%)	

* 2 × 3 Fisher's exact test; Mean [±95% CI].

During history and physical examinations, many patients described that they had complained of having frequent to daily headaches to their regular physicians. Despite this, new diagnoses were made during the clinic visit in this protocol. This may suggest under evaluation of migraines in the normal clinical setting. Treatment was initiated in about half of the GWI and CFS subjects. Subjects who developed migraines during their visit here were given trials of sumatriptan. The drug was uniformly beneficial lending additional support for migraine pathology. Results specific to these findings are reported elsewhere.

### MANOVA results

One-Way MANOVA confirmed the assumption that there would be one or more significant differences between patient groups (control, CFS, or GWI; independent variables) using the multiple study outcomes [Pillai's Trace = 0.904, *F*_(26, 212)_ = 6.73, *P* = 1.5 × 10^−16^]. The multivariate effect size (partial eta squared) was estimated at η^2^ = 0.452 which implies that 45.2% of the variance in the dependent variable was accounted for by patient grouping.

### Fibromyalgia and dolorimetry

Systemic hyperalgesia was noted as widespread pain in 62% of GWI and 70% of CFS subjects. Manual thumb-pressure causing pain at ≥11/18 tender points were more common in GWI (64%) and CFS (89%) than controls (18%; *P* = 7.7 × 10^−8^ by Fisher's Exact test). FM was diagnosed in 38 and 56% of CFS and GWI, respectively, compared to none of the control subjects. Dolorimetry indicated that GWI and CFS had significantly greater hyperalgesia over systemic regions [*F*_(2, 128)_ = 18.8, *P* = 7.5 × 10^−8^; ANOVA followed by Tukey's HSD test] and the 5 paranasal sinus regions [*F*_(2, 128)_ = 15.5, *P* = 9.6 × 10^−7^] than control groups (Table 3).

**Table 3:** **Systemic hyperalgesia**.

**Parameter**	**Controls (*n* = 45)**	**CFS (*n* = 39)**	**GWI (*n* = 50)**
Systemic dolorimetry (kg)	5.5 [4.7–6.3]	2.7 [2.2–3.0]^*^	3.5 [2.9–4.1]^*^
Sinus dolorimetry (kg)	2.2 [1.8–2.6]	0.96 [0.80–1.1]^*^	1.4 [1.2–1.7]^*^

GWI and CFS subjects had significantly lower systemic and sinus pain thresholds compared to controls.

* *P* < 0.0001 vs. controls, ANOVA followed by Tukey's test; mean [95% CI].

### Self-reported complaints

**Figure 2:**
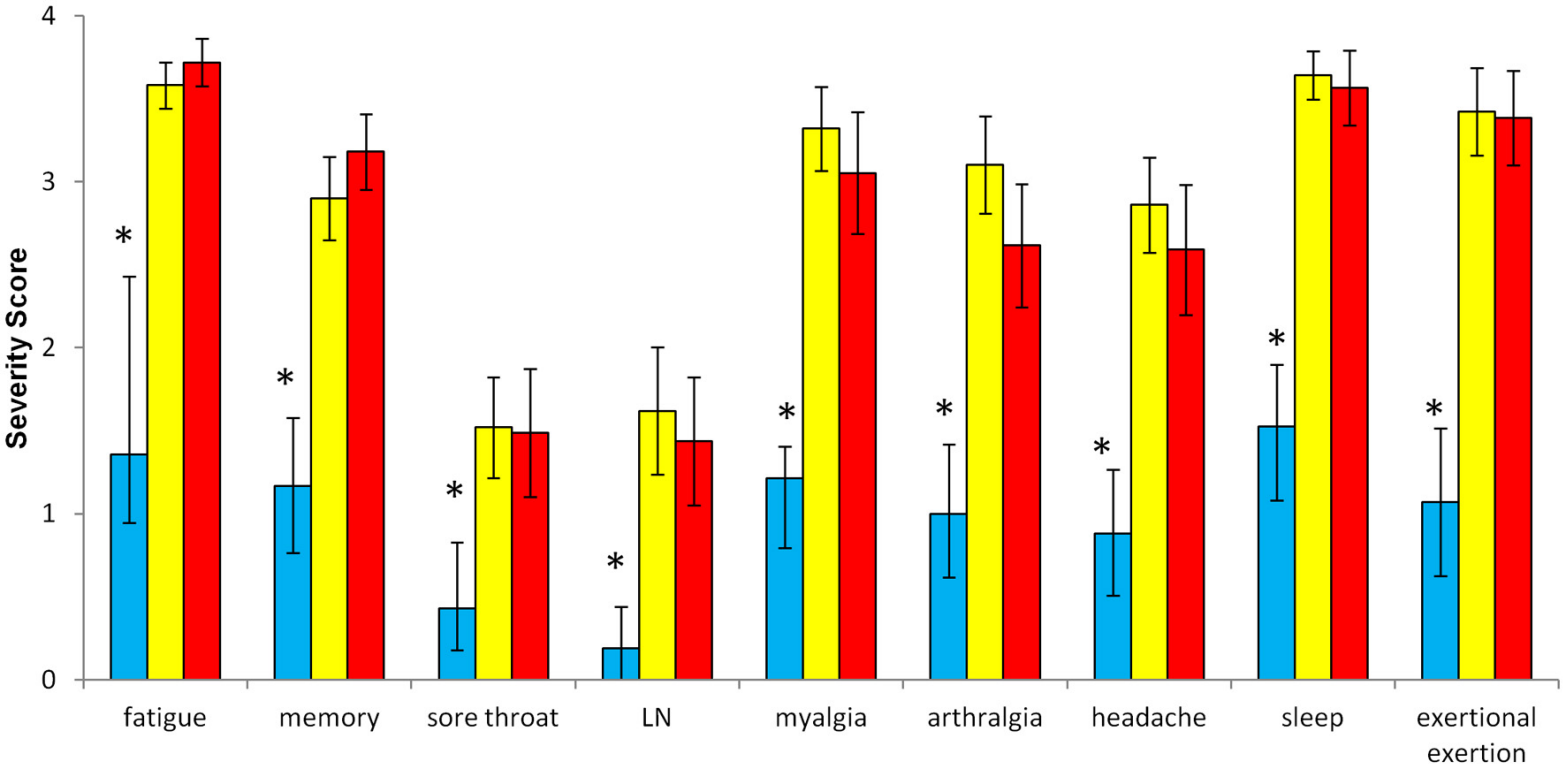
**CFS symptom severity scores.** The severity of fatigue and the 8 ancillary criteria were scored on an anchored ordinal scales from 0 to 4. Controls (blue columns) had significantly lower scores for each item compared to GWI (yellow columns) and CFS (red columns). ^*^P < 0.0000018; ANOVA followed by Tukey's HSD test; error bars depict the mean [±95% CI].

**Figure 3:**
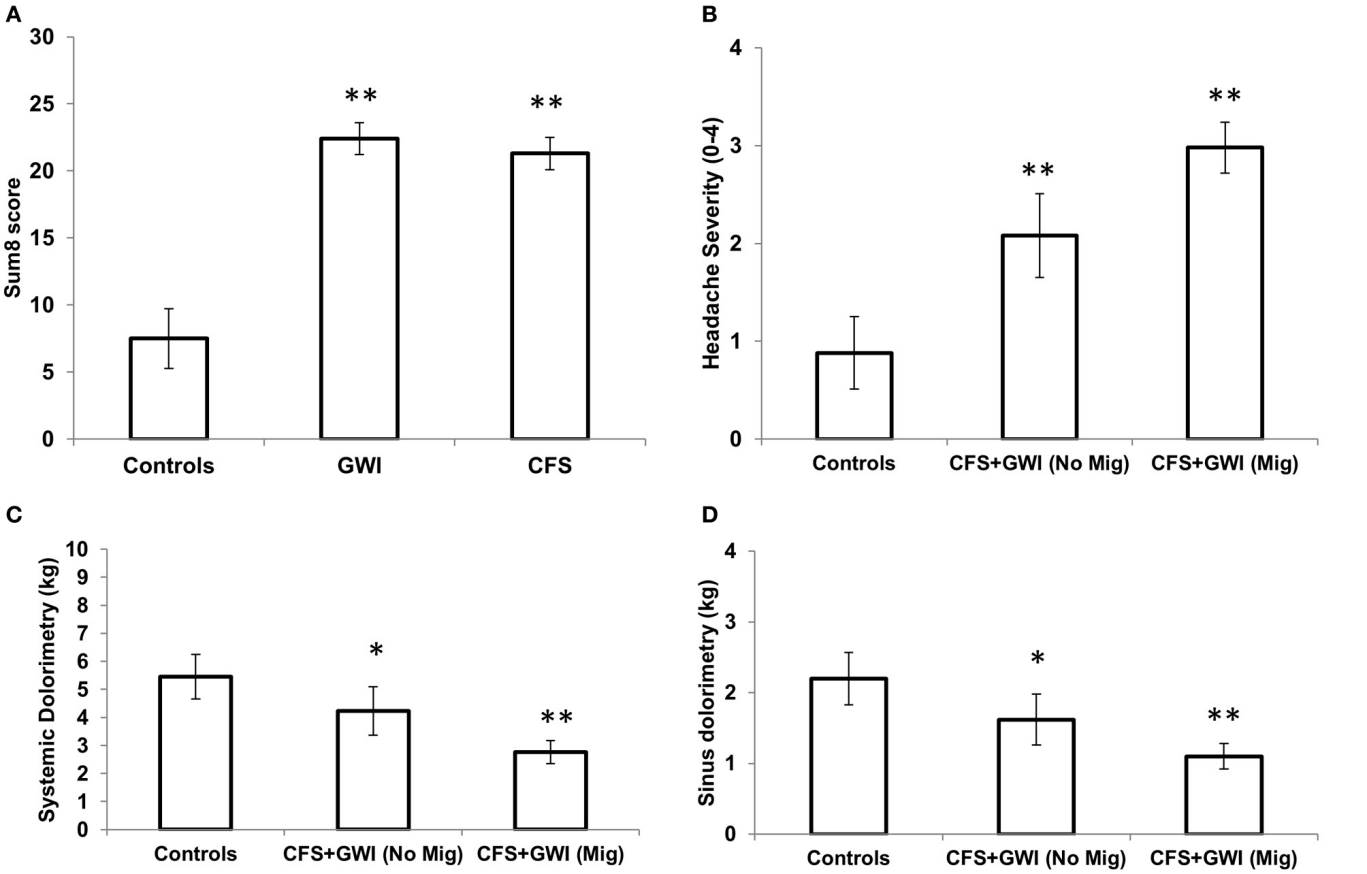
**Sum8 and comparison between CFS and GWI subjects based on migraine status. (A)** Sum of 8 scores which total the 8 ancillary criteria from the CFS severity score. **(B)** CFS and GWI subjects were combined to show that migraineurs (*n* = 64) had higher ratings of headache severity (2.9 [2.7–3.2]) compared to CFS and GWI subjects with no migraines (2.1 [1.6–2.5]; *n* = 25) and controls (0.88 [0.51–1.3]; *n* = 45). **(C)** CFS and GWI subjects with migraines had lower systemic pain thresholds (2.8 [2.4–3.2]) compared to those with no migraines (4.2 [3.4–5.1]) and controls (5.5 [1.6–2.5]). **(D)** CFS and GWI migraineurs had lower sinus pain thresholds (1.1 [0.9–1.3]) compared to CFS and GWI with no migraines (1.6 [1.3–1.9]) and controls (2.2 [1.8–2.6]). ^*^P ≤ 0.05; ANOVA followed by Tukey's HSD test; error bars depict the mean [±95% CI]. ^**^P ≤ 0.001.

The CFS severity score results were equivalent for GWI and CFS, and significantly greater than controls for fatigue, the 8 ancillary criteria, and the Sum8 score [Figures 2, 3A; *F*_(2, 128)_> 14.6, *P* < 1.8 × 10^−6^]. Sore throat and lymph nodes were the criteria with the lowest average scores in GWI and CFS. Controls had significantly lower total and subscale scores on the McGill pain questionnaire compared to CFS and GWI subjects (Table 4; *F*_(2, 128)_> 38.1, *P* < 1.2 × 10^−13^]. Controls also had higher SF-36 domain scores compared to CFS and GWI subjects [Table 4; *F*_(2, 128)_ > 40.1, *P* < 1.2 × 10^−19^].

**Table 4:** **McGill pain and SF-36 scores**.

**Parameter**	**Controls (*n* = 45)**	**CFS (*n* = 39)**	**GWI (*n* = 50)**
**MCGILL SCORE**			
Sensory domain	5.2 [3.2–7.3]	13.9 [11.6–16.1]^*^	16.2 [11.8–20.6]^*^
Affective domain	1.0 [0.47–1.5]	4.1 [3.0–4.9]^*^	6.1 [5.2–7.0]^*^
Total score	6.2 [3.7–8.7]	17.8 [15.0–20.6]^*^	23.8 [21.2–26.4]^*^
**MOS SF-36**			
Phys. Functioning	77.4 [62.9–91.8]	40.0 [33.2–46.8]^*^	43.9 [35.6–52.1]^*^
Role physical	70.6 [51.2–89.9]	7.1 [0.8–13.5]^*^	12.9 [5.1–20.6]^*^
Bodily pain	68.1 [53.7–82.5]	29.6 [23.9–35.3]^*^	28.5 [22.3–34.7]^*^
General health	67.7 [54.1–81.3]	34.7 [28.1–41.3]^*^	27.6 [22.4–32.9]^*^
Vitality	58.2 [45.7–70.7]	11.9 [7.9–15.7]^*^	16.3 [12.3–20.3]^*^
Social functioning	75.7 [61.1–90.4]	24.3 [17.9–30.7]^*^	25.0 [18.9–31.9]^*^
Role emotional	84.3 [69.4–99.3]	58.1 [43.1–73.1]^*^	30.5 [18.1–42.9]^*^
Mental health	75.3 [69.1–81.5]	64.7 [58.9–70.4]^*^	50.1 [42.3–57.3]^*^

GWI and CFS subjects self -reported significantly higher pain ratings on the McGill pain questionnaire. GWI and CFS subjects have significantly lower quality of life scores compared to controls for the eight SF-36 domains indicating impairment. (

* vs. controls, P < 0.0001; ANOVA followed by Tukey's test; Mean [±95% CI]).

### Comparison of CFS and GWI patients with and without migraine

Because there was no significant association between gender and migraines, we combined the CFS and GWI cohorts to compare groups with (*n* = 64) and without (*n* = 25) migraine diagnosis. All self-reported and demographic variables were similar. The Sum8 scores were also similar with the exception of the ordinal headache rating, systemic and sinus pain thresholds (Figures 3B–D). As expected, CFS and GWI subjects with migraines had significantly higher headache ratings compared to patients without migraines and controls [Figure 3B; *F*_(2, 128)_ = 44.4, *P* = 2.2 × 10^−15^]. In addition, migraineurs had lower systemic [Figure 3C; *F*_(2, 128 )_ = 20.9, *P* = 1.5 × 10^−8^] and sinus pain thresholds [Figure 3D; *F*_(2, 128)_ = 15.3, *P* = 1.1 × 10^−6^] indicating tenderness and potentially central sensitization.

## Discussion

All of the GWI veterans recruited met CMI and CFS criteria (Fukuda et al., 1994, 1998). Overlap in case designation criteria is consistent with previous findings (Ravindran et al., 2011; Rayhan et al., 2013a,b). Scores for self-reported questionnaires, systemic and sinus pain thresholds, and migraine for GWI and CFS subjects were comparable. The high prevalence of migraine in CFS was verified and extended to include GWI (Peres et al., 2002; Ravindran et al., 2011). GWI had significantly lower SF-36 scores than controls indicating severe impairment of quality of life (Buchwald et al., 1996). This indicates a large unmet need to diagnose and treat migraine in GWI and CFS, and to expand the options for therapy of these conditions.

GWI subjects tended to be male due to the higher ratio of men in the military. In contrast, CFS and FM is predominantly diagnosed in the civilian female population (Reiffenberger and Amundson, 1996; Okifuji et al., 1999; Eisen et al., 2005; Stephen, 2005; RAC-GWVI, 2008; Li et al., 2011). The high prevalences of migraine in GWI and CFS were independent of gender. This finding may be the result of exclusion of menstrual and chronic childhood onset migraine from our study population, but is also consistent with the lack of association of migraine with estrogen, progesterone and other sex hormone receptor polymorphisms (Schürks et al., 2010).

The causes of GWI and CFS have been hotly contested with little consensus. However, recent work suggests that toxic exposures (sarin gas, pesticides, particulates from oil fires, etc.) and/or acetylcholinesterase inhibitor use in the Persian Gulf may be temporally associated with GWI onset (RAC-GWVI, 2008; Steele et al., 2012). Diverse infectious and stressor triggers have been proposed, but not proven, to underlie CFS and FM in the civilian population (Wolfe et al., 1990; Fukuda et al., 1994; Reiffenberger and Amundson, 1996; Peres et al., 2002; Ravindran et al., 2011).

The seemingly confusing symptomatology of these disorders requires a fresh, systems biology conceptual approach that integrates systems involved in assessing afferent inputs, spinal cord and brain processing, and the conscious perceptions that are conveyed as subjective complaints. Sensory afferent information from mucosal organs such as the nose (neurological non-allergic rhinitis of CFS (Baraniuk et al., 2005), esophagus (“nutcracker esophagus”), bowel (irritable bowel syndrome) (Lea et al., 2004), bladder (irritable bladder syndrome and interstitial cystitis) is poorly localized in the insular somatosensory cortex. Interoceptive visceral sensations may enter the consciousness of some subjects and be perceived as serious illnesses (Critchley and Harrison, 2013). Chronic irritation, inflammation, or centrally released mediators may lead to excessive glutamate release that activates neuronal AMPA receptors to promote the transmission of nociceptive messages (Rowbottom et al., 1998; Goadsby et al., 2002; Fukui et al., 2008) The resulting perceptual state may be an example of neural plasticity gone awry.

Dysregulation of neuropathic sensory relays to the dorsal pons may lead to excessive pain and tenderness (hyperalgesia, allodynia), fearful memories as in posttraumatic stress disorder, impaired executive decision-making (“brain fog”), and characteristic features of GWI and CFS. The synaptic molecular changes lead to neural hypersensitivity and hyperresponsiveness to extrinsic and central, intrinsic activation that is termed “central sensitization” (Latremoliere and Woolf, 2009). A range of gene polymorphisms such as ion channelopathies may modify the risk for illnesses development in the population. For example, certain variants of Panx1 ion channels may be dysfunctional and more vulnerable to stressors that promote widespread neurotransmitter release (Rowbottom et al., 1998; Goadsby et al., 2002; Fukui et al., 2008). In a rat model of migraine, activation of Panx1 channels on peripheral trigeminal neurons caused cerebrovascular dysregulation with changes suggestive of aura followed by headache (Karatas et al., 2013). Evidence supporting central sensitization has been found in each of these chronic nociceptive, interoceptive, and fatiguing illnesses (Malick and Burstein, 2000; Maizels and Burchette, 2004; Meeus and Nijs, 2007).

Acute episodes of migraine and its consequences may provide a model for central sensitization (Figure 1). The triggering event may be an acute, extreme, and prolonged depolarization called cortical spreading depression (CSD) (Pietrobon, 2005; Smith et al., 2006; Eikermann-Haerter and Ayata, 2010). The molecular and electrical events initiating CSD involve glial and neuron cell body depolarization with an extreme efflux of K+ out of cells, and influx of Na+ and Ca2+ (Smith et al., 2006; Ayata, 2010; Charles, 2010).

Returning the intracellular K+ concentration to the high level required to repolarize these cells can take several minutes instead of milliseconds. The prolonged depolarization leads to oligemia that may cause transient hypoxia, depletion of oxygenated neuroglobin reserves, (Casado et al., 2005) and anaerobic glucose metabolism with lactic acid production. Release of autoregulatory vasodilators leads to profound reactive cortical vessel dilation and hyperemia. In animal models, the CSD spreads outwards in concentric fashion at the rate of 3–5 mm per minute as a wave of intense depolarization with oligemia followed by autoregulatory hyperemia. The change in blood flow can be detected by arterial spin labeling using 3 Tesla fMRI (T2 weighted perfusion changes) (Swartz and Kern, 2004). Peri-infarct depolarizations may be a similar phenomena in strokes, brain trauma, and other head injuries (Strong and Dardis, 2005; Fabricius et al., 2006; Strong et al., 2007). CSD may be analogous to the chaotic, stochastic cellular depolarization of atrial fibrillation, premature ventricular contractions (PVC's), esophageal spasm, or epilepsy.

Chronic CSD-like depolarization in migraine, GWI and CFS may promote central sensitization and progressive dysfunction of the anterior cingulate gyrus (ACC) and other neuroanatomical loci (Jensen et al., 2009; Obermann et al., 2009), “Neural plasticity” (Pietrobon, 2005) may reinforce conditioned memories and contribute to affective dysfunction, anxiety, fear and posttraumatic stress disorder (PTSD), (Woodward et al., 2006) fatigue, pain, hyperalgesia, allodynia and cognitive dysfunction (“brain fog”) (Meeus and Nijs, 2007). Neurovascular dysfunction may cause gray matter thinning and white matter abnormalities (prevalence = 16–40%; OR = 3.9, 95% CI = 2.26–6.72) that accentuate these disabilities (Burgmer et al., 2009; May, 2009; Schwedt and Dodick, 2009).

Alternative central sensitization hypotheses of migraine such as the “Unitary Hypothesis” suggest a primary role for cortical and limbic structures (Burstein and Jakubowski, 2005). Burstein and Jakubowski proposed that the well-known triggers of migraine stimulated multiple hypothalamic, limbic, and cortical nuclei that project axons to the superior salivatory nucleus (SSN). The SSN is a preganglionic parasympathetic nucleus that activates the postganglionic sphenopalatine ganglion. The release of acetylcholine, vasoactive intestinal peptide (VIP), and nitric oxide (NO) may initiate vasodilation of meningeal vessels. Wall stretching would then stimulate mechanoceptors on trigeminal afferent neurons that convey the signal of migraine neurovascular pain.

Another alternative is the central periaqueductal gray matter (PAG) hypothesis. The PAG has been considered a migraine generator. Positron emission tomography scans performed during spontaneous migraine attacks found activation of the PAG, dorsal pons near the locus ceruleus, and ACC (Weill et al., 1995). Activation of the ACC is not fully explained, but could indicate interference with executive decision-making and other cognitive functions (Rayhan et al., 2013a). Sumitriptan relieved the migraine symptoms, but the PAG remained activated. This suggested that the PAG may act as a “migraine generator” with the latent potential to trigger additional attacks. Other lines of evidence implicating the PAG in migraine include the correlation between the duration of illness with iron deposition (a marker of dysfunction) near the PAG, caudate and putamen (Aurora, 2009; Kruit et al., 2009). Dihydroergotamine binds to the PAG and dorsal raphe nucleus, (Goadsby and Gundlach, 1991) while naratriptan injection into the PAG has an antinociceptive effect (Bartsch et al., 2004).

Dysregulated brain stem release of NO and calcitonin gene related peptide (CGRP) may represent a molecular mechanism to initiate migraine and facilitate central sensitization show in Figure 1. These mediators may act synergistically (Panconesi et al., 2009) to initiate: (i) inhibition of anti-nociceptive functions; (ii) phantom, self-generated sensory phenomena that may include pain, visual (e.g., photosensitivity (Recober et al., 2010) auditory, and vestibular aberrations; (iii) retrograde activation of trigeminal ganglion afferents leading to CGRP release from branched nerve endings around dural and pial vessels (reverse of 3–5 on Figure 1); (iv) unregulated nociceptive responses from vascular stretch receptors (5 on Figure 1); and (v) facilitate the other ascending nociceptive and cognitive dysfunction (7–11 on Figure 1).

The “neurolimbic hypothesis” extends the role of the PAG as a “migraine generator” to include activation of a proposed neurolimbic pain network (Maizels et al., 2012). In this heuristic, migraine represents that end result of dysfunctional brain network activities, rather than the consequence of defects in individual ion channels, other proteins, or anatomical nuclei. Maizels et al. propose that brainstem pain-modulating circuits have bidirectional connections with the limbic system (amygdala, insula, ACC, orbito-frontal cortex, prefrontal cortex, hypothalamus) that interact tonically to modulate migraine expression 974).

This hypothesis is based on the existence of a major network of trigeminovascular - sensitive neurons that innervate the thalamus, with thalamic relays to widespread regions of the cortex (Noseda et al., 2011; Karatas et al., 2013). This pathway originates in the trigeminal nerve endings in pial and dural vessel walls. These primary trigeminal neurons innervate the cell bodies of second-order trigeminovascular neurons that are located in laminae I and V of the dorsal horn of the caudal medulla and upper cervical C1 and C2 spinal segments. The secondary ascending neurons have been traced electrophysiologically to the midbrain, where they bifurcate into an axon projecting to thalamic posterior (Po), ventral posteromedial (VPM), and parafascicular nuclei, and a second axonal limb that arborizes and terminates in anterior, lateral, perifornical and posterior hypothalamic nuclei, and lateral preoptic areas. The third-order trigeminovascular—responsive thalamic neurons convey signals to wide variety of cerebral regions. VPM and Po neurons have axons that bifurcate and issue dense terminal arborations in the trigeminal barrel-field region of the primary somatosensory cortex (S1BF) and secondary somatosensory cortex (S2). Additional Po neurons project to extra-trigeminal regions of S1, the primary auditory (AuD/Au1), retrosplenial (RSA), parietal association (PtA), primary and secondary visual (V1/V2) cortices, and primary and secondary motor cortices (M1/M2). Neurons from the laterodorsal (LD) and lateroposterior (LP) thalamic nucleus project to M1 and M2 regions, trigeminal and extratrigeminal S1 regions, secondary visual cortex mediomedial (V2MM), and the ectorhinal cortex (Aurora, 2009; Burstein et al., 2010; Noseda et al., 2011). This pathway connects the primary meningeal nociceptive signals to the functionally distinct and anatomically remote cortical areas that are involved in migraine—related dysregulation of affect, motor function, visual and auditory perception, spatial orientation, memory retrieval, olfaction, and allodynia (Burstein et al., 2010).

Resting state fMRI functional connectivity studies of interictal migraine subjects provide additional support for abnormal brainstem—limbic system interactions (Mainero et al., 2011). Functional connectivity is the property of brain regions to harmoniously signal to each other, and indicates their capability to act in cooperative fashion. Migraineurs had increased functional connectivity between the PAG and thalamus, posterior parietal cortex, anterior insula, somatosensory cortex that are involved in somatosensory, interoceptive, and nociceptive processing. Those subjects with a high frequency of migraines had increased connectivity between the PAG, anterior insula, nucleus cuneiformis, and hypothalamus, but reduced connectivity of PAG with the prefrontal cortex, ACC, amygdala, and medial thalamus. Decreased connectivity between the PAG and anterior and posterior cingulate cortex, hippocampus, putamen, and posterior insula has been confirmed in a separate group who had frequent migraines (Maleki et al., 2011a). Women had more significantly reduced functional connectivity of the PAG to the posterior cingulate cortex and amygdala than men (Maleki et al., 2011b). Functional connectivity alterations were more pronounced in adults than children (Lebel et al., 2011). When allodynia was present, migraineurs had decreased connectivity between the PAG, prefrontal cortex, ACC, and anterior insula. Therefore, magnetic imaging studies in GWI and CFS should take into account potential changes in baseline cerebrovascular hemodynamic responses (Rayhan et al., 2013b), gray matter interconnectivity, and white matter integrity (Rayhan et al., 2013a). Disruption of these intricate neural pathways may represent a new paradigm for understanding complex neurological diseases.

These objective findings have important clinical implications for the subjective presentation of migraine, GWI and CFS subjects. A common presentation is:

“… a middle-aged woman with chronic migraine and medication overuse, as well as fibromyalgia. In addition, there is anxiety and depression, fatigue and insomnia, and the familiar exhaustive list of psychotropics and antiepileptic drugs tried and failed” (Maizels and Burchette, 2004).

This type of patient may seem exceedingly frustrating, since many physicians find the panoply of symptoms daunting and their armamentarium bare. The physician's quandary can be deepened as they investigate the history further. Regardless of whether they consider themselves “lumpers” or “splitters,” the presence of one functional syndrome increases the probability of others (Bradley, 2008). The situation is all the more exasperating since current medical dogma does not provide a satisfactory explanation for the underlying pathophysiology. Maizels et al. (2012) provide a logical approach based on their neurolimbic model, knowledge of the dysfunctional of serotonergic neural pathways, and central nature of the nociceptive, interoceptive, somatosensory, and fatigue complaints (Table 5).

**Table 5:** **Approach to patients with overlapping migraine, GWI, CFS, FM, and other disorders (adapted from Maizels et al., 2012)**.

**Intervention**	**Benefit**
Clinical evaluation	Evaluate personality style (e.g., perfectionism, caregiver), lifestyle factors (“pressure cooker”), life circumstances (e.g., an abusive relationship), other stressors, coping skills, and psychiatric comorbidity that have a profoundly negative impact on quality of life.
Patient education	Limbic functions regulate mood, emotion, perceptions, and responses to stressors, personality, coping styles, and cognitive function.	
	These have beneficial and detrimental effects on affect, fatigue and pain.
	Depression and anxiety are important risk factors for migraine transformation (Lipton, 2009). Affective dysfunction should be anticipated with this complex of unpredictable complaints and current unsatisfactory treatment options.
Physician education	Conceptualizing these illnesses as dysfunctional pain, interoceptive, cognitive, and other brain networks is novel, and more difficult to appreciate than a specific genetic, neurotransmitter, or brainstem nucleus problem.	
	This realization opens the way to diagnosis, lays a solid foundation for the patient–doctor relationship and expands the scope of therapeutic options.
Treatment	Although there is not as yet evidence to show that treating psychiatric comorbidity influences headache outcomes, it appears clinically prudent to do so.
	Non-pharmacologic treatments such as cognitive behavioral therapy, carefully prescribed activity levels, acupuncture, tai chi, and other efforts to “retrain the brain” are often neglected aspects of treatment.
	Combinations of low dose pharmaceutical, physical, cognitive, and other therapies are likely to be superior to single modalitie (Holroyd et al., 2009, 2010).
Research	Atypical responses to stressors will play a critical role in the identification of biomarkers, neuroimaging characteristics, future diagnostic algorithms, pathophysiological mechanisms, and logical and beneficial treatments.
	The impact of triptan drugs, topiramate, and other migraine therapies on GWI and CFS functional status has not been studied.
Limitations	Identification of unrecognized exacerbating factors, inadequate non-pharmacologic treatment, and the presence of comorbid conditions such as anxiety, pain, and migraine is an essential component of building a satisfactory healthful relationship (Lipton et al., 2003).
	Set attainable limits and goals for cognitive and physical activities, and expectations of benefits from current medical treatments.
	Regular follow-up can ensure treatment compliance and reinforce the security of on-going clinical care without the perception of abandonment or rejection felt by many GWI and CFS patients.

This cross-sectional investigation had several limitations. Although presence and absence of aura was identified, during subgroup analysis of migraines subjects were not grouped based upon the presence or absence of aura due to the small sample size. It was not designed to correlate migraine phenotypes in GWI with triggers such as fasting, loss of sleep, odors, hormonal changes, and stress (Fukui et al., 2008). Longitudinal changes in migraine severity, systemic hyperalgesia, fatigue, and functional status await larger epidemiological and treatment studies. Analysis of magnetic imaging correlates are pending.

## Conclusion

GWI and CFS subjects have a high prevalence of migraine headaches, comparable patterns of systemic and cognitive complaints, and share overlapping phenomenological case designation criteria. CFS and GWI had lower systemic pain thresholds indicating systemic hyperalgesia and central sensitization. The loss of descending inhibitory antinociceptive aminergic signals may contribute to spinal sensitization, but cannot explain the many other cerebral, brainstem, and autonomic abnormalities. It is tempting to speculate that the parallel findings of GWI, CFS, and migraine indicate a shared underlying pathophysiological mechanism of central nervous system neural pathways that may account for chronic pain, fatigue, and cognitive dysfunction (“brain fog”). Interdisciplinary studies that address these diverse components of illness are required to identify relevant dysfunctional pathways and molecular mechanisms that may lead to reductionist approaches for evaluation of future drug treatments.

## Authors' contributions

James N. Baraniuk and Murugan K. Ravindran designed the protocol. Rakib U. Rayhan, Murugan K. Ravindran, and James N. Baraniuk administered and conducted the study. Rakib U. Rayhan and James N. Baraniuk interpreted and completed the statistical analysis. Rakib U. Rayhan and James N. Baraniuk wrote, edited, and gave final approval for the manuscript.

### Conflict of interest statement

The authors declare that the research was conducted in the absence of any commercial or financial relationships that could be construed as a potential conflict of interest.
